# Peri-implant health is associated with low levels of TNF-α

**DOI:** 10.1590/0103-644020256205

**Published:** 2025-10-24

**Authors:** Juliana Prazeres Gonçalves de Castro, Valquiria Quinelato, Luiza dos Anjos Motta, Telma Regina da Silva Aguiar, Aldir Nascimento Machado, Priscila Ladeira Casado

**Affiliations:** 1 Department of Implant Dentistry Post-graduation, Fluminense Federal University- School of Dentistry- Niterói- RJ- Brazil

**Keywords:** Periimplant health, biomarkers, periimplant treatment, TNF Alpha, periimplant mucositis

## Abstract

This study aimed to assess tumor necrosis factor-alpha (TNF-α) expression in peri-implant crevicular fluid (PICF) of patients receiving peri-implant supportive therapy. Twenty-six participants with implant-supported prostheses, functioning for at least 1 year, were divided into two groups: control (peri-implant health, n=8) and mucositis (peri-implant mucositis, n=18). All underwent clinical and radiographic exams and received non-invasive therapy (mechanical debridement, with ultrasonic scalers to remove calculus and sodium air-abrasive polishing to eliminate soft debris from the implant and prosthetic components + 0.12% chlorhexidine). Timepoints were: Time 1 (initial diagnosis/treatment); Time 2 (15 days post-diagnosis); Time 3 (21 days after). PICF samples collected at each time point analyzed TNF-α levels via ELISA. While clinical parameters showed no group differences, a trend (p=0.07) towards more keratinized gingiva in controls was noted. Significant TNF-α differences were found over time in the control group (p = 0.029, Kruskal-Wallis), with Dunn's test showing T2/T3 differences (p=0.03). The Mucositis group showed no time differences (p=0.96). Over 36 days, no significant difference (p=0.86, Kruskal-Wallis) was found between the control and mucositis groups. TNF-α levels decreased significantly after 36 days in both groups, more so in controls. Therapy reduced mucositis from 70% to 23%. Overall, while TNF-α levels alone may not conclusively differentiate between healthy and mucositis-affected sites in every context, they do appear to correlate with improvements in peri-implant health following therapeutic intervention.

## Resumo

Este estudo teve como objetivo avaliar a expressão do fator de necrose tumoral alfa (TNF-α) no fluido crevicular peri-implantar (FCPI) de pacientes recebendo terapia de suporte. Vinte e seis participantes com próteses suportadas por implantes, em funcionamento por pelo menos 1 ano, foram divididos em dois grupos: controle (saúde peri-implantar) e mucosite (mucosite peri-implantar). Todos foram submetidos a exames clínicos e radiográficos e receberam terapia não invasiva (desbridamento mecânico + clorexidina a 0,12%). Os pontos temporais foram: Tempo 1 (diagnóstico/tratamento inicial); Tempo 2 (15 dias após o diagnóstico); Tempo 3 (21 dias depois). Amostras de FCPI coletadas em cada tempo foram analisadas quanto aos níveis de TNF-α via ELISA. Embora os parâmetros clínicos não tenham mostrado diferenças entre os grupos, foi observada uma tendência (p=0,07) para mais gengiva queratinizada nos controles. Diferenças significativas nos níveis de TNF-α ao longo do tempo foram encontradas no grupo controle (p = 0,029, Kruskal-Wallis), com o teste de Dunn mostrando diferenças entre T2/T3 (p=0,03). O grupo mucosite não mostrou diferenças ao longo do tempo (p=0,96). Ao longo de 36 dias, não foi encontrada diferença significativa (p=0,86, Kruskal-Wallis) entre os grupos controle e mucosite. Os níveis de TNF-α diminuíram significativamente após 36 dias em ambos os grupos, mais acentuadamente nos controles. A terapia reduziu a mucosite de 70% para 23%. TNF-α poderia ser um alvo futuro no tratamento da mucosite, pois níveis mais baixos indicam melhor saúde peri-implantar.

**Figure:**
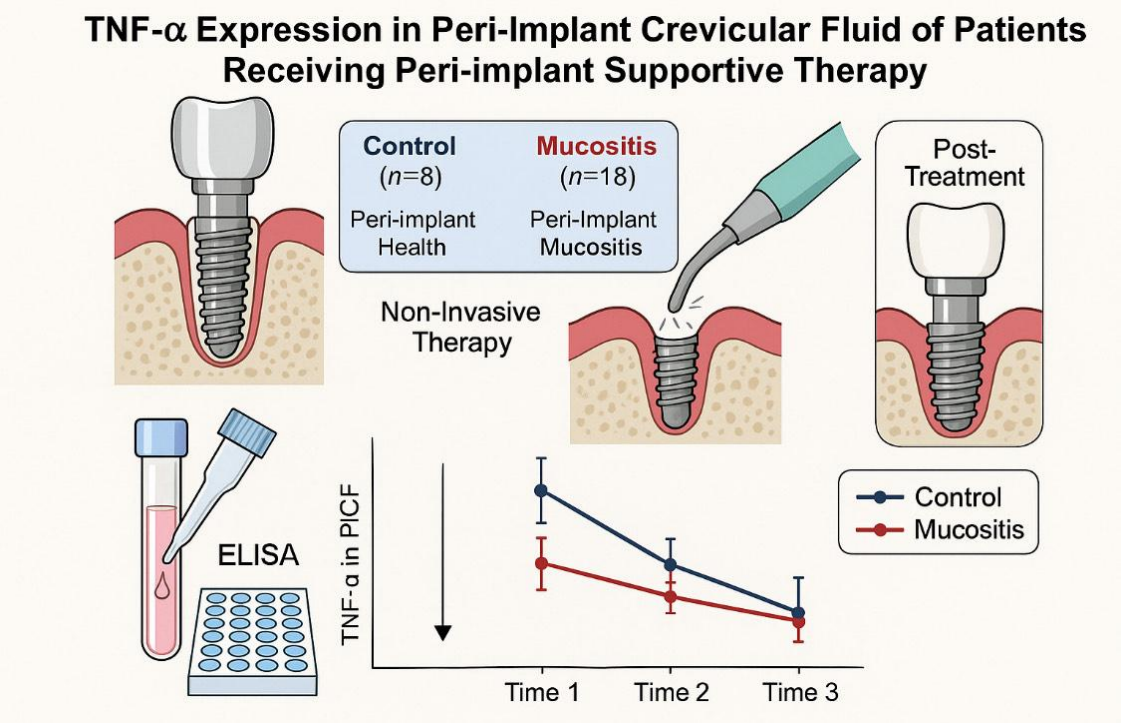

## Introduction

Peri-implant disease is a multifactorial disease characterized by the presence of inflammation in both soft tissues (mucositis) and hard tissues (peri-implantitis) around the implant ^1^. Its etiopathogenesis is related to numerous intrinsic and extrinsic factors that, together, can trigger an exacerbated inflammatory response around the implant, culminating in peri-implant bone resorption and even implant loss ^2^.

According to Group 1 of the Consensus conducted in 2019 by the FDI (World Dental Federation) ^3^, the prevalence of peri-implant mucositis is 43%, and peri-implantitis 22%, becoming the treatment with endosseous implants, once highly predictable, susceptible to failure.

Considering the diagnosis, clinical methods should be used to detect the presence or absence of inflammation at the implant site, including visual inspection, peri-implant probing, and palpation. Probing of peri-implant tissues is necessary to assess bleeding and monitor clinical depth, observing changes and migration of mucosal margins. These evaluations can alert the clinician to the need for therapeutic intervention ^4^.

Peri-implant health is characterized by the absence of erythema, bleeding, swelling, and suppuration, as well as the absence of pathologic bone loss ^5^. Differently, mucositis is a common inflammatory condition that affects the soft tissues surrounding dental implants. It can progress to peri-implantitis, leading to implant failure if not managed timely. The pathogenesis of peri-implant mucositis involves a complex interplay between microbial factors and the host's immune response. Among the various cytokines implicated in the inflammatory response, Tumor Necrosis Factor-alpha (TNF-α) has garnered significant attention due to its pivotal role in orchestrating inflammatory processes ^6^.

TNF-α is a pro-inflammatory cytokine that mediates a wide range of cellular responses, including apoptosis, cell proliferation, and the expression of other inflammatory mediators ^7^. Elevated levels of TNF-α have been observed in various oral inflammatory conditions, suggesting its potential involvement in the pathophysiology of peri-implant mucositis ^6^.

In addition, it is presumed that peri-implant mucositis is the precursor of peri-implantitis, similar to how gingivitis precedes periodontitis. Therefore, the prevention of peri-implant diseases involves preventing peri-implant mucositis and converting peri-implant mucositis to health ^1^.

The absence of annual supportive therapy in patients diagnosed with peri-implant mucositis is associated with an increased risk of conversion from mucositis to peri-implantitis. According to Costa et al. ^8^ after 5 years, 18% of patients who adhered to peri-implant supportive therapy were diagnosed with peri-implantitis, compared to the incidence of 43.9% of peri-implantitis in patients not undergoing supportive therapy.

Levels of biochemical mediators secreted in PICF have been considered diagnostic markers for monitoring health ^9^, reflecting the degree of inflammatory and regenerative reaction affecting the surrounding tissues, bone, and mucosa ^10^. Researchers have correlated biochemical markers of inflammation and regeneration with clinical parameters observed in the peri-implant region, in health and disease ^11^ ^,^ ^12^ ^,^ ^13^.

The identification of biomarkers around the implant during mucositis treatment can elucidate the local response to peri-implant therapies. Understanding the correlation between TNF-α levels and peri-implant mucositis can provide insights into the mechanisms underlying this condition and inform the development of targeted diagnostic and therapeutic strategies. Therefore, the present study aimed to analyze the expression of TNF-Alpha in the PICF of patients undergoing peri-implant support therapy. We hypothesize that elevated TNF-α levels are correlated with peri-implant mucositis, which could suggest a potential biomarker for early detection and management of this condition. By studying the expression of TNF-α in peri-implant mucositis, we hope to contribute to the growing body of research aimed at improving patient outcomes and ensuring the longevity of dental implants.

## Material and methods

This is a non-randomized cross-sectional study approved by the Research Ethics Committee from Antônio Pedro University Hospital / Fluminense Federal University, under number 2.455.991. The clinical data was collected in the Implant Dentistry Department of Fluminense Federal University and the laboratory assays were carried out in the Clinical Research Unit of the Antonio Pedro University Hospital.

### Inclusion Criteria

 As far as the authors know, this study included healthy patients, with and without a previous history of periodontitis. The participants were partially edentulous patients with previous radiography showing the alveolar bone level around teeth, with an external hexagon implant installed for at least 1 year finished with fixed total Branemark’s protocol prostheses in the mandible and who signed the free and informed consent form.

### Exclusion Criteria

The excluded participants were those with systemic diseases (diabetes, blood dyscrasias), smokers, and those who take resorptive drugs and hormones. Patients who underwent mouthwash, antibiotic, and/or anti-inflammatory therapy 3 months prior to the research and those diagnosed with peri-implantitis or who underwent supportive peri-implant therapy 6 months before research diagnosis were also excluded.

### Participants

The study included all patients who visited the Implant Dentistry Department of Fluminense Federal University between 2018 and 2020 and received an external hexagon dental implant, along with fixed total Branemark’s protocol prostheses in the mandible. Out of 42 initial patients, 4 were excluded due to systemic diseases, 6 because of smoking, 3 due to relocation, and 3 diagnosed with peri-implantitis who were referred for specialized treatment in the Implant Dentistry Department. Consequently, 26 participants were chosen for the study.

### Clinical Examination and Treatment

A single investigator (J.P.G.C.), calibrated using the kappa-test (Kappa value 0.92), conducted the clinical examination, sample collection, and treatment. The implant-supported prostheses were removed, and all implants and peri-implant tissues were examined at three different time points: Time 1 (initial clinical and radiographic examination/diagnosis); Time 2 (15 days after initial treatment); Time 3 (21 days after the second treatment). The peri-implant clinical examination involved assessing the mesial, distal, buccal, and lingual surfaces of each implant using a North Carolina periodontal probe (PCP-UNC 15, Hu-Friedy Manufacturing Inc., Chicago, IL, USA). Additionally, the distance between the prosthetic ridge and peri-implant mucosa was evaluated using a straight-tip specimeter (Golgran- Millennium, São Paulo, SP, Brazil).

Peri-implant health and mucositis were diagnosed based on the parameters outlined in the 2019 Diagnosis and Non-Surgical Treatment of Peri-Implant guidelines. Initially (Time 1), participants were categorized into two groups: the control group (n=8), characterized by good peri-implant health for all implants, and the mucositis group (n=18), characterized by peri-implant mucositis in at least one implant. During re-evaluation (Times 2 and 3), participants could be reclassified into either the control or mucositis group based on new diagnoses. Consequently, participants from the mucositis group could be included in the control group if their peri-implant health conditions improved.

### Radiographic Evaluation Before Implant Placement

The examination aimed to detect the presence or absence of alveolar bone resorption around the teeth. The determination of a history of periodontitis was based on the criteria outlined by Caton et al.^14^ and was part of the clinical protocol at the University.

### Radiographic Evaluation During Peri-Implant Diagnosis

Another author (T.A.), as a single operator performed the digital radiographic examination around the implant to identify the peri-implant bone level and possible peri-implanttis diagnosis. This exam consisted of periapical digital radiographs (Indicator Digital Shick Elite, KODAK RVG5100 Digital Radiography System - São José dos Campos, SP-Brazil) using a positioner (Indusbello Londrina, PR-Brazil) performed in the RX device (DABI ATLANTE Spectro 70x RX, Ribeirão Preto, SP-Brazil). For diagnostic reasons, the radiographic examination was performed on all participants at Time 1. When participants were diagnosed with peri-implantitis, they were excluded from this study and referred to a supportive program in the Implant Dentistry Department. The physiological bone loss was characterized considering the normal bone loss of one millimeter during the first year after implant placement and of 0.2 mm per subsequent year according to the period of osseointegration^15^. The crestal bone loss calculation was based on the difference between the radiographic examination at baseline (considering external hexagon implant placement at the bone level for all included implants) and the radiographic aspect during supportive therapy. From these parameters, when the total bone loss was higher than expected (1 mm in the first year and 0.2 mm in subsequent years under loading conditions)^15^, the diagnosis was peri-implantitis, taking into account the pathologic bone loss.

### Treatment Protocol

All participants in this study underwent the same treatment regimen, irrespective of their diagnosis. Ultrasonic scalers (Cavflex 6000 Dentflex®, Ribeirão Preto, SP, Brazil) were utilized for calculus removal. In the control group (healthy mucosa), ultrasonic scalers were used to remove calculus from the prosthesis and supragingival calculus that did not affect mucosal health. Sodium air-abrasive polishing (JetlaxisUNO Schuster, Santa Maria, RS, Brazil) was then employed to eliminate soft debris from the implant, prosthetic components, and fixed implant-supported prostheses (Table 1). Participants underwent the same protocol because, regardless of calculus accumulation in peri-implant tissue or implant-supported prostheses, necessitating removal, the presence or absence of peri-implant inflammation (mucositis) was observed.

**Table 1:** Summary of treatment protocol performed at each interval.

Intervals	Treatment Protocol
TIME 1: Diagnosis	- Ultrasonic scaler/ Air polishing
	- Mouthwash with chlorhexidine (2x/day- 14 days)
	- Prosthetic repair
	- Instruction on self-performed plaque removal
TIME 2: 15 days after time 1	- Ultrasonic scaler / Air polishing
TIME 3: 21 days after time 2	- Ultrasonic scaler / Air polishing

Oral and written instructions for self-performed plaque control were provided to the participants. Prosthetic adjustments were made to facilitate hygiene access around the implants. All implant-supported fixed protocols were removed, cleaned, and polished using tungsten carbide drills (Série Ultra 079KFF - Dhpro - Paranaguá, Paraná - Brazil), finishing and polishing points (Kit PA10203Dhpro - Paranaguá, PR - Brazil), and were immediately replaced. Prior to treatment, peri-implant crevicular fluid (PICF) was collected at the specified intervals mentioned previously. Debris around the implants was cleared with cotton pellets, and a paper tip filter (Maillefer - Dentsply, Petrópolis, RJ-Brazil) was inserted into the peri-implant crevicular region for 60 seconds, then immediately submerged in 400μl of PBS/1% BSA (fetal bovine serum/bovine serum albumin) and stored at -80°C (Froi Lab Ultra Low Temperature DW HL678S - São Paulo, SP - Brazil).

For sample collection, the site with the highest degree of disease for each participant was selected. In cases where all peri-implant regions were healthy, the implant site nearest to the midline was chosen for collection. Twenty-six samples were collected at each time point, totaling 78 samples collected throughout the course of this research.

### Tumor Necrosis Factor Alpha Levels in the Peri-Implant Crevicular Fluid

TNF-A levels in the samples were determined by enzyme-linked immunosorbent assay (ELISA) using the Human TNF Alpha PicoKineTM ELISA Kit (900-K08 - Peprotech, Rocky Hill, NJ, USA). Analyzes of the diluted samples were performed by following the manufacturer's recommendations. The readings were made in 110 μL of the sample, with filters of 405 nm wavelength, generated from a standard curve of concentrations. After mathematical conversion, considering the dilution, the standard curve, and the dispersion graphic, the TNF-A concentrations were defined in pg / µl.

### Statistical Analysis

Data processing and statistical analysis were conducted using Prism GraphPad software version 6.0 (GraphPad Software, La Jolla, CA, USA). The distribution of numerical variables was assessed using the Shapiro-Wilk test. Student's t-test or Mann-Whitney's test was employed for normally or non-normally distributed data, respectively. The chi-square test was utilized with a significance level of 0.05 (p < 0.05) to correlate peri-implant mucositis with nominal clinical variables. The risk of developing peri-implant mucositis was evaluated in relation to nominal variables using odds ratios (OR), with a 95% confidence interval constructed accordingly.

## Results

### Clinical Results

Out of the initial 42 volunteers evaluated over a period of 1 year, 26 were included in this research. Among them, there were 16 (61.5%) men and 10 (38.5%) women, with a mean age of 65.5 ± 7.99 years.

At baseline, there was no significant difference observed between the control group and the mucositis group regarding gender, age, and history of periodontitis (Table 2).

During the primary diagnosis (Time 1) participants were divided into 2 groups: Control (n=8) and Mucositis (n=18), showing no difference in clinically evaluated parameters in peri-implant sites, prostheses, and implants. However, there was a tendency of association (p=0.07) between a higher range of keratinized gingiva in the control group.

**Table 2:** Clinical aspects of all participants: Time 1.

PARAMETERS	CONTROL (n=8/%)	MUCOSITIS (n=18/%)	p-value (OR; CI)*
GENDER			
MALE	3 (37.5)	13 (72.2)	0.10 (0.23: 0.03-1.34)
FEMALE	5 (62.5)	5 (27.8)	
AGE	67.75±2.1	64.72±2.0	0.38
PERIODONTITIS HISTORY	5 (62.5)	8 (44.4)	0.33 (2.08: 0.37-11.48)

.*OR: odds ratio; CI: confidence interval

After initial treatment (15 days, Time 2) and final treatment (36 days from baseline, Time 3), the control group and mucositis group showed a difference in the width of keratinized mucosa (p=0.03) at Time 3. The presence of a greater width of keratinized mucosa was significantly associated with healthy peri-implant sites (control group).

**Figure 1:**
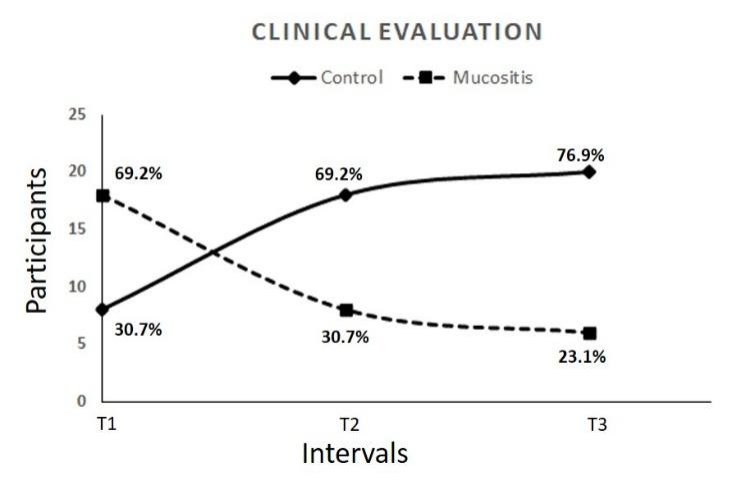
Clinical Evaluation from Time 1 to Time 3, after 36 days of mucositis treatment and supportive therapy (p=0.03).

However, the transition of participants from the mucositis group to the control group was evident after initial supportive therapy, during Time 2 (control=18; mucositis=8), and at Time 3 (control=20; mucositis=6). After 36 days, the clinical mucositis incidence decreased from 70% to 23%, showing significant improvement after primary treatment (Time 1) (p=0.003) Figure 1.

### Laboratory Results (TNF-A Levels)

**Figure 2:**
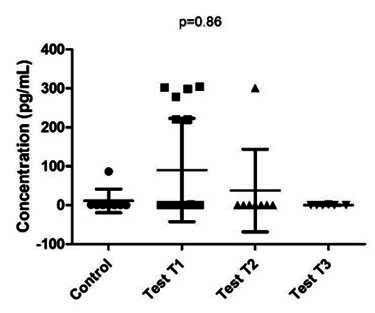
Comparison between test groups (peri-implantar mucositis) and control (no disease) three times. (T1 without treatment); T2 (15 days after mechanical debridement) and T3 (30 days after mechanical debridement). No statistical difference have been between the groups p=0.86 (Kruskal-Wallis test). * (Control group = no disease in T1).

Considering the comparison between the Control and Mucositis groups throughout the 36 days (all treatment intervals), there was no statistically significant difference in TNF-A levels (p=0.86) (Figure 2).

**Figure 3:**
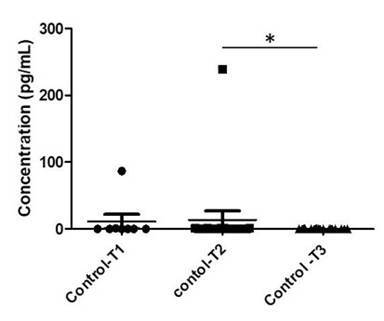
TNF-A levels comparison among intervals in the control group. (T1 without treatment (mechanical debridement)); T2 (15 days after mechanical debridment) and T3 (36 days after mechanical debridement). There were statistical differences among intervals, p=0.029 (Kruskal-Wallis test). The Dunn's Multiple Comparison Test showed the difference between T2 and T3, p=0.03.

**Figure 4:**
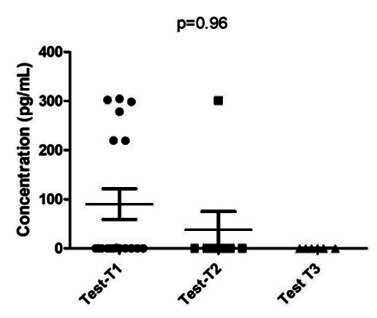
TNF-A levels comparison among intervals in mucositis group. There were no statistical differences among intervals (Kruskal-Wallis test; p=0.96).

The analysis comparing each interval (T1, T2, and T3) within each group separately revealed differences among each time point in the Control Group (p=0.03) (Figure 3). There was a significant decrease in TNF-A levels in PICF from the control group, showing that the decrease of TNF-A levels is associated with health aspects. However, there was no difference observed in the Mucositis Group (Figure 4).

## Discussion

Pro-inflammatory cytokines are among the most investigated biomarkers in peri-implant regions, playing a significant role in inflammation. Their importance, coupled with clinical data, aims to prevent and provide a deeper understanding of the pathogenesis of peri-implant disease ^16^.

The aim of this study was to analyze the expression of TNF-Alpha in the PICF of patients undergoing peri-implant support therapy. Our results provide important insights into the dynamics of peri-implant mucositis treatment and the role of TNF-α as a potential biomarker. TNF-A was clearly associated with the healthy peri-implant condition, clinically.

In peri-implantitis, IL-6, IL-1β, TNF-α, MMP-8, and their genetic variations appear to be the most important cytokines not only in pathogenesis but also in their potential diagnostic capabilities. Epithelial and inflammatory cells, along with those of the bone lineage, are the main cellular elements found in the disease. Thus, a wide variety of cells are involved in peri-implant disease, as well as cytokines and their genetic variations that participate in the process. However, the growing interest in this topic has led to the introduction of new specific diagnostic tools to enable a better understanding of patient responses to treatment and, in turn, to even predict the risk of developing peri-implant disease ^17^. The analysis of cytokine levels can help describe the pathogenesis and early diagnosis of peri-implantitis, providing a strong prediction in high-risk patients ^18^.

In our study, interestingly, despite the significant clinical improvements, no statistically significant differences in TNF-α levels were observed between the Control and Mucositis groups across the 36 days. This lack of difference could indicate that TNF-α, while important in the inflammatory response, might not be a sensitive indicator of mucositis severity when comparing between groups.

More intriguingly, within-group analyses revealed significant differences in TNF-α levels at different time points only in the Control group. The control group experienced a noticeable decrease in TNF-α levels over the treatment period, suggesting that lower TNF-α levels are associated with healthier peri-implant conditions. This decrease in TNF-α levels in the control group could reflect the stabilization or resolution of an inflammatory process, further aligning TNF-α as a marker of peri-implant health rather than a marker of disease severity.

Conversely, the Mucositis group did not show significant changes in TNF-α levels across the different time points. This finding might be attributed to the chronic nature of inflammation in mucositis, where TNF-α levels may remain elevated or fluctuate less noticeably due to continuous, low-grade inflammation that does not significantly alter across short intervention periods. It suggests that while TNF-α is relevant in the inflammatory process, its levels alone may not be sufficient to distinguish between different stages or severities of peri-implant mucositis without considering other clinical and inflammatory markers. However, in physiological situations, moderate expression of TNF-α is necessary for the maintenance of low-grade inflammation and normal implant osseointegration ^19^. This is consistent with our study, where the analysis of the comparison among each interval (T1, T2, and T3) within each group separately showed differences among each time point in the Control Group.

Gleiznys et al. ^20^ evaluated the correlation of non-surgical treatment of mucositis and its association with clinical parameters and local patterns of osteoimmune-inflammatory mediators (IL-17 and TNF-α) and matrix metalloproteinase-8 (MMP-8) in peri-implant crevicular fluid. The levels of TNF-α in peri-implant crevicular fluid samples showed a moderate correlation with plaque index, in the Mucositis group. Thus, concluding that the evaluation of inflammatory cytokine levels in the fluid may assist in identifying peri-implant mucositis, which can aid in early diagnosis, prevention, and treatment. The levels of TNF-α and IL-17 were significantly lower in patients in the treated group compared to peri-implant mucositis before treatment, which corroborates with our study, as treatment progress resulted in a reduction of TNF-α. Although peri-implant mucositis therapy is considered necessary to prevent peri-implantitis, professionals should focus on preventing mucositis, considering findings that during the 21-day plaque accumulation period, the gums around teeth develop a weaker inflammatory response compared to soft tissues around implants ^21^.

Gomes et al.^22^, after evaluating the levels of salivary biomarkers IL-1β, IL-10, RANK, OPG, MMP-2, TG-β, and TNF-α in individuals diagnosed with peri-implant mucositis in the absence or presence of periodontal and peri-implant maintenance therapy, over 5 years, observed a higher incidence of peri-implantitis in the group that did not receive therapy (43.9%) compared to the treated group (18%). The study results also revealed an increase in salivary TNF-α concentration in the untreated group compared to the therapeutically treated group, showing a beneficial role of maintenance therapy in balancing the periodontal and peri-implant clinical condition, which corroborated with our research when there was a transition of participants from mucositis group to control group after initial supportive therapy, during Time 2 (control= 18; mucositis=8), and at Time 3 (control=20; mucositis=6). The transition observed after the initial supportive therapy, where participants from the mucositis group moved towards the control group during Times 2 and 3, suggests the effectiveness of the treatment administered. The substantial reduction in clinical mucositis incidence from 70% to 23% over 36 days highlights the positive impact of early intervention and supportive therapy in reversing or mitigating the progression of peri-implant mucositis.

Recent research^23^ regarding the importance of keratinized tissue around implants in preventing peri-implant diseases and its comparison with TNF-α parameters showed that despite there being no difference in TNF-α levels between wide and narrow keratinized tissue, there was a Strong association between healthy clinical parameters and the width of keratinized tissue of the peri-implant tissue, indicating the importance of keratinized tissue around the implant in maintaining gingival health and preventing peri-implant diseases. In our study, after initial treatment (Time 2) and at the final treatment (Time 3), the significance in the width of keratinized mucosa at Time 3 underscores the importance of keratinized tissue in maintaining peri-implant health. The control group exhibited a greater width of keratinized mucosa, which was notably associated with healthy peri-implant sites. This reinforces the clinical importance of maintaining or augmenting keratinized mucosa around implants, as supported by other studies ^24^ ^,^ ^25^.

This study underscores the clinical significance of keratinized mucosa in peri-implant health and the potential of TNF-α as a biomarker for peri-implant condition monitoring. The marked improvement in clinical parameters with reduced mucositis incidence post-treatment highlights the effectiveness of early and supportive therapy. Future research should focus on long-term studies and investigate additional inflammatory biomarkers to provide a more comprehensive understanding of the inflammatory processes in peri-implant diseases. It would also be beneficial to explore the potential synergistic roles of other cytokines and inflammatory mediators alongside TNF-α to identify a more sensitive and specific biomarker panel for peri-implant mucositis. One of the limitations of the study is the initial number of participants in the control group. Certainly, replicating this study with a larger sample will further enhance the understanding of translational studies in the treatment of peri-implant mucositis. Overall, while TNF-α levels alone may not conclusively differentiate between healthy and mucositis-affected sites in every context, they do appear to correlate with improvements in peri-implant health following therapeutic intervention. The continuation of efforts to integrate clinical, laboratory, and possibly genetic data will be crucial for advancing diagnostics and tailored treatments in implant dentistry.
